# Surgical Scar Recurrence of Bone Metastases to the Femur: A Case Report

**DOI:** 10.7759/cureus.3385

**Published:** 2018-09-29

**Authors:** Marc Gaudet, Kelly Linden, Jean-Michel Caudrelier, Kristopher Dennis

**Affiliations:** 1 Radiation Oncology, The Ottawa Hospital & University of Ottawa, Ottawa, CAN

**Keywords:** skin, scar, recurrence, cancer, bone metastases, post-operative, radiotherapy

## Abstract

We report the case of a woman who presented with breast cancer metastases to the femur causing pathologic fracture of the femoral neck requiring surgery. She received adjuvant radiotherapy to the femur at that time that did not include the surgical scar tract. Almost four years after her surgery she presented with biopsy proven skin recurrence of breast cancer on the skin overlying her incision from her femoral surgery.

Further imaging confirmed significant soft-tissue disease involving the underlying surgical scar tract. This case provides important information about the possibility of surgical scar recurrence after surgery for bone metastases which could indicate the need to include the area of the surgical scar tract and the entire prosthetic material in the post-operative radiotherapy volume.

## Introduction

Bone metastases are present in up to two-thirds of patients with metastatic cancer [1]. The primary elements of treatment of symptomatic bone metastases include adequate analgesia and palliative radiotherapy. Nonetheless, certain clinical scenarios including pathological fracture, impending fracture or neurological compromise require consideration of surgical intervention. Post-operative radiotherapy is indicated in the majority of these cases as it treats residual disease, likely improves functional status and decreases the need for further orthopedic procedures [2, 3]. Nonetheless, there is a paucity of information regarding the anatomical patterns of relapse and progression of bone metastases other than those in the spine to help guide treatment planning of radiotherapy [4, 5]. One lingering question regarding target volumes for palliative radiotherapy is whether the operative bed and surgical scar should be included in radiotherapy volumes in addition to areas of bone that were involved preoperatively. We present the interesting case of an 81-year-old woman who presented with relapse of breast cancer in her femur both in her surgical scar and at the margin of her previous radiotherapy field.

## Case presentation

The patient was diagnosed in 2004 at age 67 with a pT2 (4.8 cm) N1 (1/15 axillary nodes) grade two lobular carcinoma of the breast and was treated with modified radical mastectomy. Her initial tumor was estrogen receptor positive and progesterone receptor negative. She was then subsequently treated with adjuvant FAC (5-Fluorouracil, Adriamycin, Cyclophosphamide) chemotherapy followed by adjuvant radiotherapy to the chest wall and supraclavicular lymph node region. She then received Tamoxifen for two and a half years, then received Exemestane for one year which she tolerated poorly and was put back on Tamoxifen to complete the balance of a five-year course of hormonal therapy.

**Figure 1:**
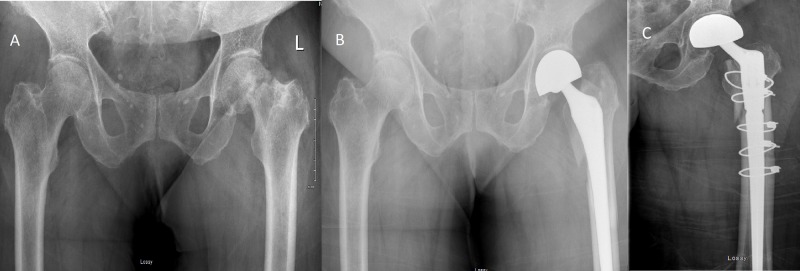
Plain anterior-posterior (AP-PA) radiographs showing initial pathological fracture (A), first surgical intervention with early periprosthetic fracture (B) and result of revision surgery (C).

**Figure 2:**
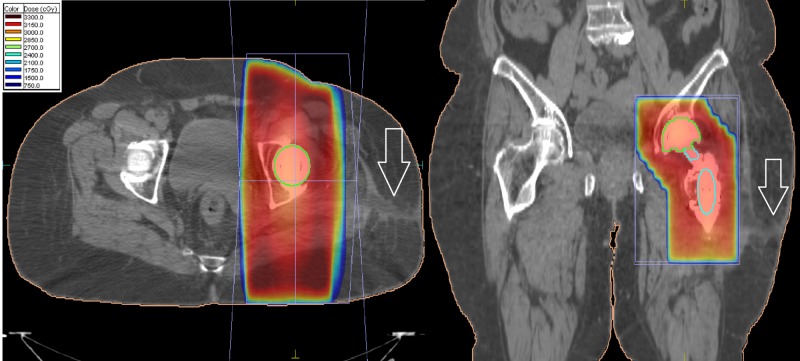
Post-operative radiotherapy at time of initial diagnosis of left femur metastases. Note surgical scar tract not covered by radiation field. The radiation field covered the area of pathologic fracture but did not include inferior extent of prosthetic material.

She showed no evidence of disease until 2014 when she presented with a pathological fracture of her left femoral neck. Figure 1A shows imaging of her plain X-rays at time of pathological fracture. This was treated surgically with a left bipolar hip hemiarthroplasty through a lateral Hardinge approach (Figure 1B). Pathology from this initial surgery confirmed the presence of metastatic adenocarcinoma consistent with her initial breast primary. Unfortunately, three days post-operatively, she suffered a fall and a periprosthetic fracture that required revision surgery with cerclage which used the same lateral Hardinge incision (Figure 1C). Chest, abdominal and whole-body bone imaging confirmed no other sites of metastases. She then received radiotherapy to the left proximal femur to a dose of 30 Gy in 10 fractions with an AP-PA technique as shown in Figure 2. This did not include the entirety of the prosthetic hardware, nor did it include the surgical scar tract within the soft tissues of the hip (as shown by arrow in Figure 2). There was no documented severe toxicity from her radiotherapy treatments. She was started on pamidronate which she continued until 2018. No other systemic treatment was given at that time because of the fact she had no other evidence of disease and patient preference.

**Figure 3:**
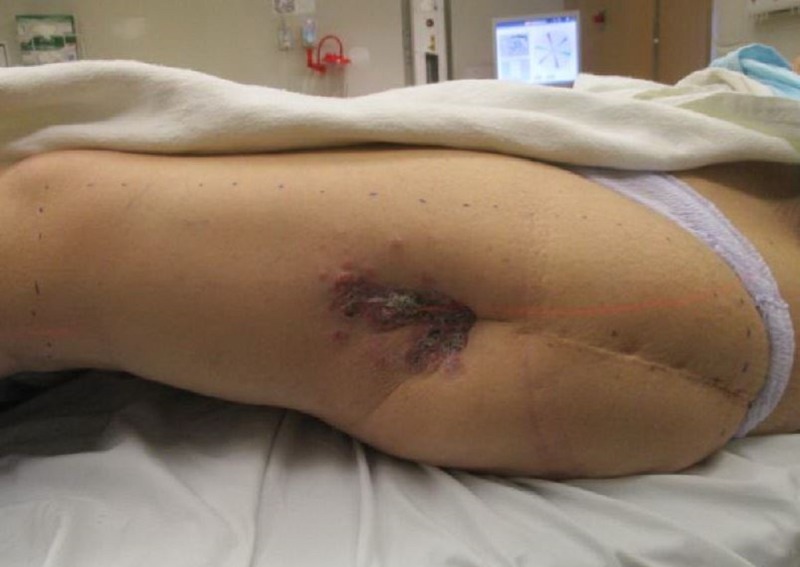
Multiple metastatic skin nodules at site of previous incision.

**Figure 4:**
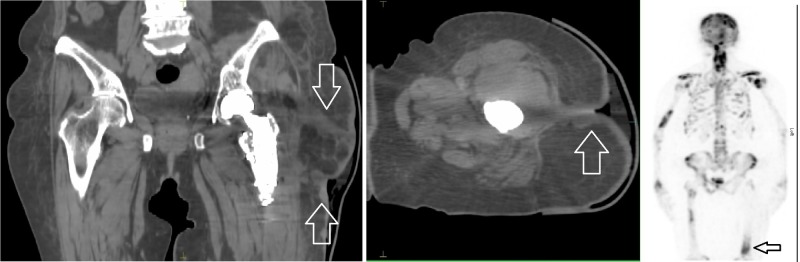
Coronal (left) and axial (centre) computed tomography (CT) scan images showing area of recurrence tracking along surgical scar site (white arrows) and bone scan (right) showing distal femur progression below previous radiation field (black arrow).

**Figure 5:**
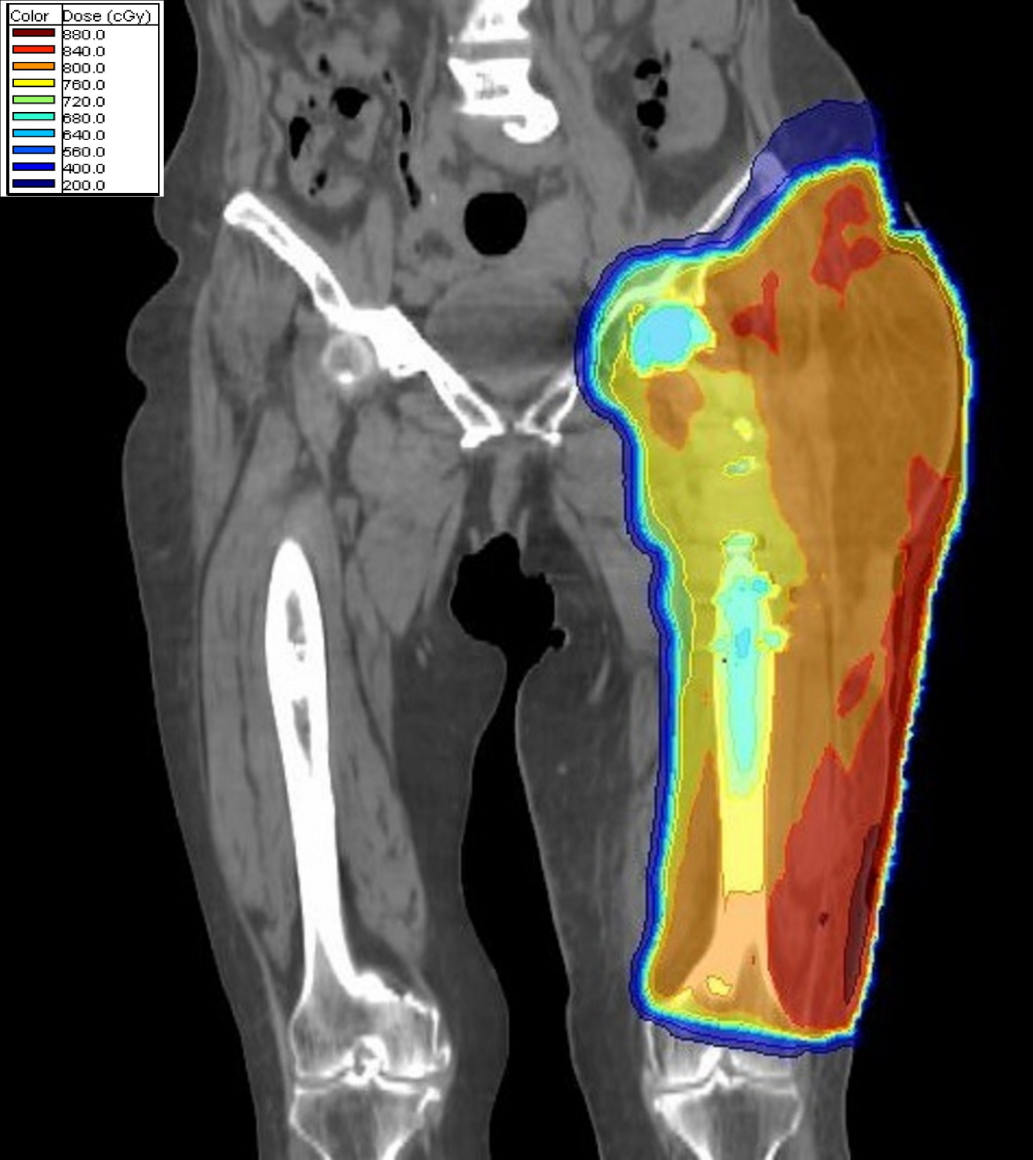
Dosimetry from radiation given at time of recurrence including treatment involving the skin and site of surgical scar recurrence.

She presented with progressive disease in multiple bony sites (ribs, sacrum, skull, lumbar spine) and liver in 2016. Progressive uptake on bone scan was noted starting in late 2016 in both the area of her previous left femoral surgery and the mid-shaft of the left femur immediately distal to the prosthetic material in place. She was treated sequentially with multiples lines of systemic therapy including Tamoxifen, Fulvestrant and Capecitabine with continued progression. In February 2018 she developed skin nodules overlying the previous incision site on left lateral thigh which were initially treated as a herpes zoster infection. These lesions progressed to become nodular and ulcerated. A biopsy of this area was done which was compatible with skin metastases from her breast carcinoma. Figure 3 shows skin changes overlying her surgical incision site at the time of radiotherapy. Computed tomography (CT) scan of this area confirmed quite extensive soft tissue extension along the tract of her surgical scar tract that extended out to the skin (Figure 4) as well as progressive bone disease at the distal edge of her previous radiation field on bone scan as shown in Figure 5. She was then treated with palliative radiotherapy of a single fraction of 8 Gray (Gy) to this area because of poor performance status. At the time of treatment, the patient was planned with CT simulation with wax bolus to smooth areas of skin depression in the area of the surgical scar. Bolus with a thickness of 0.5 cm was used over the entire area with visible or palpable skin nodules and the entire length of the scar. Clinical target volume (CTV) for the retreatment included the entire left femur from the femoral head to the distal femoral body, including laterally out to the skin surface where there were visible skin changes. The knee joint was excluded from the treatment field. Planning target volume (PTV) was created by expanding the CTV by 1 cm. The radiation plan was generated with a source skin distance (SSD) of 110 cm with parallel-opposed 6 Megavolt (MV) photon fields to allow adequate coverage (Figure 5).

## Discussion

Surgical tract and procedure site recurrences have been reported in many different cancers [6-8]. It has also been shown that relatively low dose radiation could prevent recurrence in these sites [7]. However, there is very little in the literature with regards to surgical site recurrence in cases of orthopedic surgery for bone metastases. Although surgical scar recurrences have been anecdotally reported by many oncologists, we believe this is the first formally reported case of surgical scar recurrence after surgery for bone metastases. There are many peculiarities to this patient’s case that could explain an increased risk of this type of recurrence; she did require two surgical interventions at a very short time interval which did not include any formal debulking of tumor; she also has a very prolonged history of metastatic breast cancer with this recurrence happening almost four years after her initial femur surgery and post-operative radiotherapy. Furthermore, this area was not included in her post-operative radiotherapy volume when she received adjuvant radiation to the femur.

**Figure 6:**
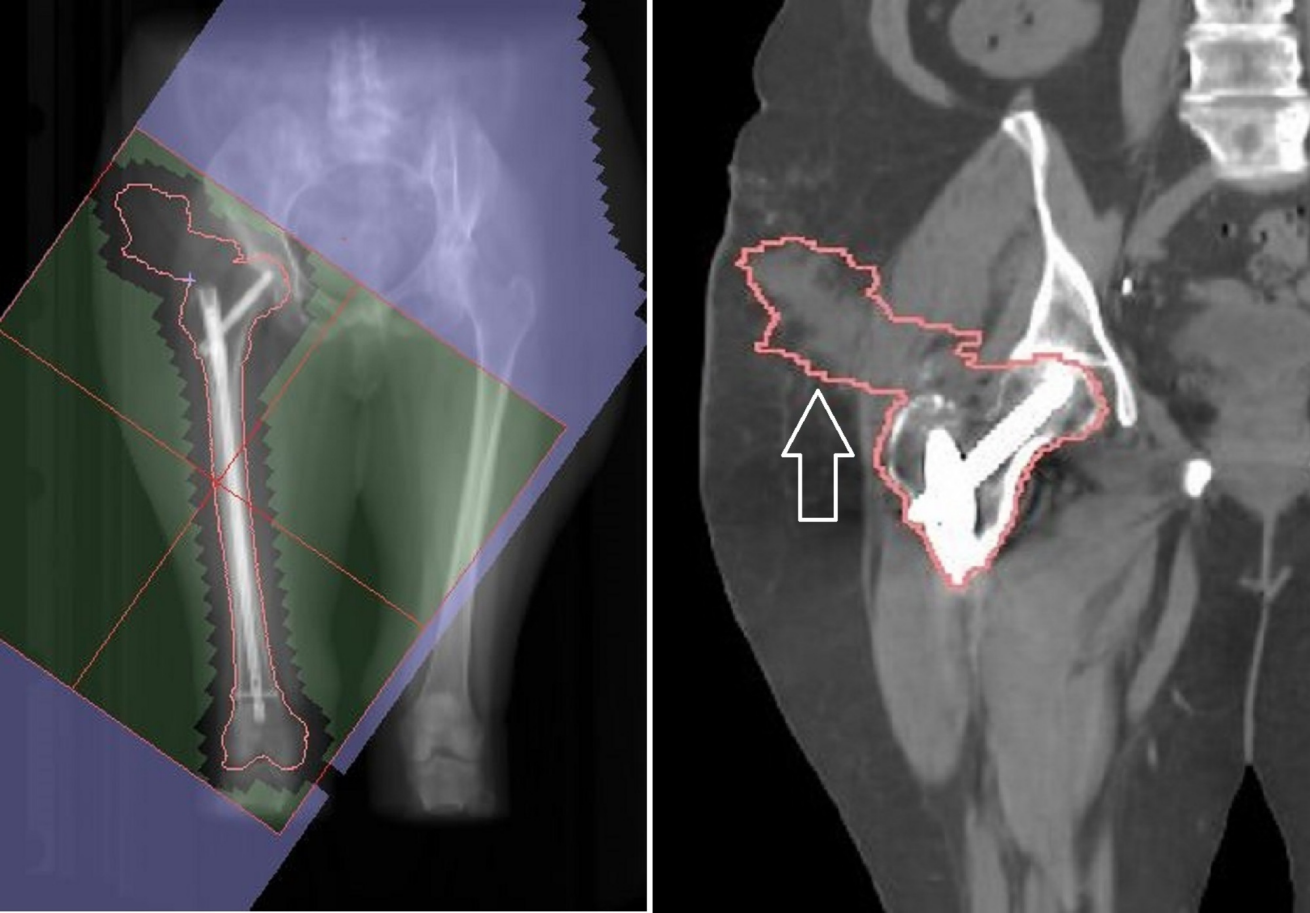
Digitally reconstructed radiograph (DRR) showing suggested volume and typical field (outlined in green) for post-operative radiotherapy after surgical intervention to the femur for bone metastases. Note clinical target volume (CTV) extending along surgical scar tract (white arrow) and treatment of entire prosthetic material.

The major learning point from her case is related to the natural history of bone metastases to long bones post-operatively. Many reports have shown the fact that adjuvant radiotherapy in these cases can reduce progressive disease, improve quality of life and decrease the need for further orthopedic procedures to the same area [2, 3]. One could hypothesize that if the area of this patient’s surgical scar tract had been included in her initial radiation field, she might not have recurred in this fashion. Guidelines for the treatment of bone metastases with conventional radiotherapy mention very little with regards to target volumes for palliative radiotherapy in general; even less guidance is provided as to post-operative volumes other than for spine radiotherapy [5, 9, 10]. Classic dogma would state that one should include the entire operative bed and prosthetic material, but this is not always the case in daily practice. More recent evidence from stereotactic body radiotherapy (SBRT) for bone metastases in the pelvis provides some evidence for wider treatment fields, as most areas of progressive disease in pelvic bones can appear 15-55 mm out of field [11]. Our current approach in our institutional dedicated rapid palliative radiotherapy service involves treating at minimum involved area of bone, plus the entirety of prosthetic material (if not the entire involved bone) as well as the surgical scar tract out to the skin (Figure 6). With palliative radiotherapy, one must be mindful to treat only volume deemed necessary not to increase toxicity in an unneeded fashion; however, in cases of bone metastases treated surgically, we do not feel that including a wider margin of soft tissue out to the skin would substantially increase toxicity with doses used for bone metastases treatment. Despite low incidence of scar recurrences, we believe these notions should be considered for incorporation into further iterations of international guidelines on radiotherapy for bone metastases.

## Conclusions

This case illustrates a previously underreported phenomenon of surgical scar recurrence of breast cancer metastases after surgical intervention to the femur. We believe this information can be useful in guiding radiation oncologists in the determination of treatment volumes in cases of post-operative radiotherapy for non-spinal bone metastases.

## References

[1] Coleman RE (2006). Clinical features of metastatic bone disease and risk of skeletal morbidity. Clin Cancer Res.

[2] Townsend PW, Smalley SR, Cozad SC, Rosenthal HG, Hassanein RE (1995). Role of postoperative radiation therapy after stabilization of fractures caused by metastatic disease. Int J Radiat Oncol Biol Phys.

[3] Drost L, Ganesh V, Wan BA (2017). Efficacy of postoperative radiation treatment for bone metastases in the extremities. Radiother Oncol.

[4] Redmond KJ, Lo SS, Soltys SG (2017). Consensus guidelines for postoperative stereotactic body radiation therapy for spinal metastases: results of an international survey. J Neurosurg Spine.

[5] Redmond KJ, Robertson S, Lo SS (2017). Consensus contouring guidelines for postoperative stereotactic body radiation therapy for metastatic solid tumor malignancies to the spine. Int J Radiat Oncol Biol Phys.

[6] Song J, Kim E, Mobley J (2014). Port site metastasis after surgery for renal cell carcinoma: harbinger of future metastasis. J Urol.

[7] O'Rourke N, Garcia JC, Paul J, Lawless C, McMenemin R, Hill JA (2007). Randomised controlled trial of intervention site radiotherapy in malignant pleural mesothelioma. Radiother Oncol.

[8] Stigliano R, Marelli L, Yu D, Davies N, Patch D, Burroughs AK (2007). Seeding following percutaneous diagnostic and therapeutic approaches for hepatocellular carcinoma. What is the risk and the outcome? Seeding risk for percutaneous approach of HCC. Cancer Treat Rev.

[9] Lutz S, Balboni T, Jones J (2017). Palliative radiation therapy for bone metastases: update of an ASTRO evidence-based guideline. Pract Radiat Oncol.

[10] Lutz S, Berk L, Chang E (2011). Palliative radiotherapy for bone metastases: an ASTRO evidence-based guideline. Int J Radiat Oncol Biol Phys.

[11] Ito K, Shimizuguchi T, Nihei K (2018). Patterns of intraosseous recurrence after stereotactic body radiation therapy for coxal bone metastasis. Int J Radiat Oncol Biol Phys.

